# Meditative Movement, Energetic, and Physical Analyses of Three Qigong Exercises: Unification of Eastern and Western Mechanistic Exercise Theory

**DOI:** 10.3390/medicines4040069

**Published:** 2017-09-23

**Authors:** Penelope Klein, George Picard, Joseph Baumgarden, Roger Schneider

**Affiliations:** 1 Physical Therapy Department, D’Youville College, Buffalo, NY 14201, USA; george@georgepicard.com (G.P.); jbaumgard2016@gmail.com (J.B.); 2 Village of Healing and Wellness, St Catharines, ON L2R 3L2, Canada; rschnei487@aol.com

**Keywords:** Qigong, Tai Chi, Meditative Movement, Theory, Movement Analysis, Exercise

## Abstract

Qigong is the meditative movement and therapeutic exercise of Eastern medicine. A growing body of evidence is validating its health benefits leading to mechanistic questions of how it works. The purpose of this article is to explore mechanisms of action related to Qigong, with the intent of unifying Eastern and Western exercise theory and to present a model for Qigong exercise analysis. Three exercises from a standardized Qigong form: ‘Plucking the Stars’, ‘Lotus Leaves Rustle in the Wind’, and ‘Pacing Forwards and Backwards’ were selected for meditative, energetic, and physical analyses. Meditative aspects include relaxation response, interoception and exteroception. Energetic aspects include stimulation of meridians through mental intent, acupressure, and self-massage. Physical aspects include flexibility, strength, articular stimulation, neuro-integration, respiratory effect, fascial stretch, visceral massage, balance challenge CranioSacral pump, lymphatic and venous return and glandular stimulation, and physiologic response to relaxation. Knowledge of mechanisms of action for specific Qigong exercises can guide operational definition of Qigong, selection of outcomes assessment in future research, inform prescriptive practice addressing clinical health issues, and advance adoption of Qigong practice within integrative health care. The model of analysis demonstrated in this discussion may assist in these endeavors.

## 1. Introduction

Qigong is a broad discipline within Chinese philosophy. Academic study is composed of essential theories, practical skills, and clinical applications [1]. Areas of study include spiritual exploration and development, martial arts, and medical therapy [2]. Medical Qigong dates back over 5000 years, and its practice endures today within Traditional Chinese Medicine (TCM) and more recently within modern integrative health care. Basic tenets of practice are rooted in Yin/Yang and 5 Element Theory. Qi, as vital life energy, accumulates and circulates through the body to provide nutritional support for optimal health [1]. An applied definition of Qigong explains Qigong as the adjustment of body, breath, and mind transcending into oneness [1]. In TCM, disease is attributed to excess or deficiency of Qi resulting in disruption of the flow of Qi to systems, organs and structures. When one is in a state of Qi or life energy sufficiency and balance, one is considered to be in a state of optimal health. A clinical goal of Medical Qigong is to maintain or restore the natural balance and flow of Qi and its associated nutritive benefits. There are two major sources of Qi: non-renewal Qi, also known as prenatal or original Qi, and renewable Qi that which is derived from food, air, nature, and among other sources, meditation and mindful exercise [3]. This applied theoretical discussion is an exploration of the mechanics of cultivation of renewable Qi through internal processes related to practice of Qigong and Tai Chi exercise as performed for health.

A consensus working paper (2005) on Qigong and Tai Chi, generated by clinical researchers and experts in the field, identified the essential elements of Qigong practice to be either static or dynamic exercise combined with breath regulation, mental intent, and self-massage [4]. Self-massage techniques include tapping along meridians and energy gates as well as bone marrow washing (tapping along bones): stroking or combing of the biofield (the energetic field that exists within and surrounding the physical body); foot massage through weight shifting; fascial compression of organs and soft tissue during stretching movement; even vocalizations of healing sounds creating as internal vibratory force. 

### 1.1 Study Rationale

In 2016, a collaborative bibliometric review identified over 500 studies investigating therapeutic benefits of Tai Chi [5]. Researhers concluded that the quantity and strength of evidence is substantial. However, given the lack of intervention definition and wide variation in Tai Chi/Qigong forms studied, further well-designed trials are recommended to better define protocols and the mechanisms of action for mind/body exercise. While research can inform on matters of effectiveness, applied theory is needed to explain mechanisms of action. 

### 1.2. Clinical Research Evidence

A recently published map of 107 systematic reviews assessing benefits of Qigong exercise identified a wide range of outcome measures studied [6]. Two thirds of the reviews were published in the last 5 years. Clinical areas with the strongest evidence base, as validated in systematic reviews, meta-analyses, and key independent studies are balance and fall prevention [7,8,9], supportive cancer care [10,11,12,13,14], supportive cardiac care [15,16,17,18], and alternative rehab management of Chronic Obstructive Pulmonary Disease [19,20]. There is moderate- to low-level evidence validating Qigong practice as complementary in management of Parkinson’s disease [21,22], fatigue [23,24], and fibromyalgia [25,26]. There is also exploratory-level evidence suggesting benefit in other clinical areas [6,27,28]. 

### 1.3. Theoretical and Conceptual Framework

The following addresses the theoretical and conceptual framework of the current discussion. It is organized into three major foci: (a) meditative movement as a collective construct, (b) application of TCM Energy Theory, and (c) physical aspects of Qigong practice consistent with a Western medicine approach. 

### 1.4. Meditative Movement (MM)

Similar to conclusions of the 2005 Expert Panel on Qigong and Tai Chi, Larkey and colleagues propose that essential characteristics of MM consist of: first, a meditative state of mind, usually involving a focus of awareness on the body; second, some form of prescribed (or sometimes spontaneous) movement; third, explicit attention to the breathing; and fourth, a state of deep relaxation [29]. Practices within the collective construct known as MM include Taijiquan, Qigong, and Hatha (postural) Yoga, as well as modern Western modalities such as the Alexander Technique, and Feldenkrais. 

Relaxation responses are integral to MM practice. Payne et al., addressed the concept that relaxation responses maintain a state of completely balanced tone, eutonis, in which every muscle is doing exactly what it should [30]. This state is experienced as light, free, open, and effortless; but at the same time stable, powerful, and well-rooted. Dr. Herbert Benson, a noted pioneer in the field of relaxation response, maintains that eliciting a state of relaxation by calming the mind combined with slow deep breathing and focusing mental attention can easily be learned [31]. In the context of healing, he uses the term: remembered health, as key to recruiting endogenous mechanisms for self-restoration of health. Slow, deep breathing with calming mental intent, as practiced in Qigong, has been shown to increase parasympathetic activity in both healthy and symptomatic populations [32,33]. In a related discussion, Sawynok proposed that Qigong, as a self-practice, leads to enhanced parasympathetic activity, and this underlies benefits in management of fibromyalgia and contributes to other health benefits that occur with extended practice [34].

This relaxation response leads to decreases in heart rate and blood pressure, release of muscle tension, improved digestion, improved sleep cycles, enhanced anti-inflammatory effects, decreases in stress and anxiety, decreased pain, enhanced mood, and helps to regulate the autonomic nervous system [31]. However, MM is more than just relaxation. The mind/body experience originates through the integration of interoceptive (internal physiological sense of body-ownership) and exteroception (interpretation of external inputs obtained through sources such as touch and vision) [35]. This complex system of heightened sensing primary within MM including Qigong practice aids in formation of a deeper understanding of self. 

### 1.5. Energy Theory

Foundational within Energy Theory are at least two contributory theories. Yin/Yang Theory and 5 Element Theory. Yin/Yang Theory predicates on an understanding that optimal function (health, sense of well being, and harmony) results from a balancing of opposite natural forces and their respective interplay (e.g., dark/light, soft/hard, feminine/masculine) [3]. Similar to the balance of opposites with no extremes is the balance of the five elements. In TCM, the five elements are identified as fire, earth, metal, water, and wood [3]. Each element links to specific organ function: fire: heart, pericardium, small intestine, and triple warmer; earth: stomach, spleen/pancreas, metal: lungs, large intestine; water: kidneys and urinary bladder; and wood: liver and gall bladder. These elements and their associated organs interrelate in constructive and regulatory cycles [3]. For example: in its constructive cycle, wood feeds fire; in its regulatory cycle, wood penetrates earth. As with Yin/Yang theory, it is the interdependent balancing of the five elements and their inherent properties that is thought to promote optimal organ function and health. Knowledge of the interdependence of the five elements can guide all aspects of life including dietary choices. In TCM, the quest to achieve balance and harmony in life and Qi cultivation is advocated as the path to optimal health, healing and longevity. 

**Figure 1:**
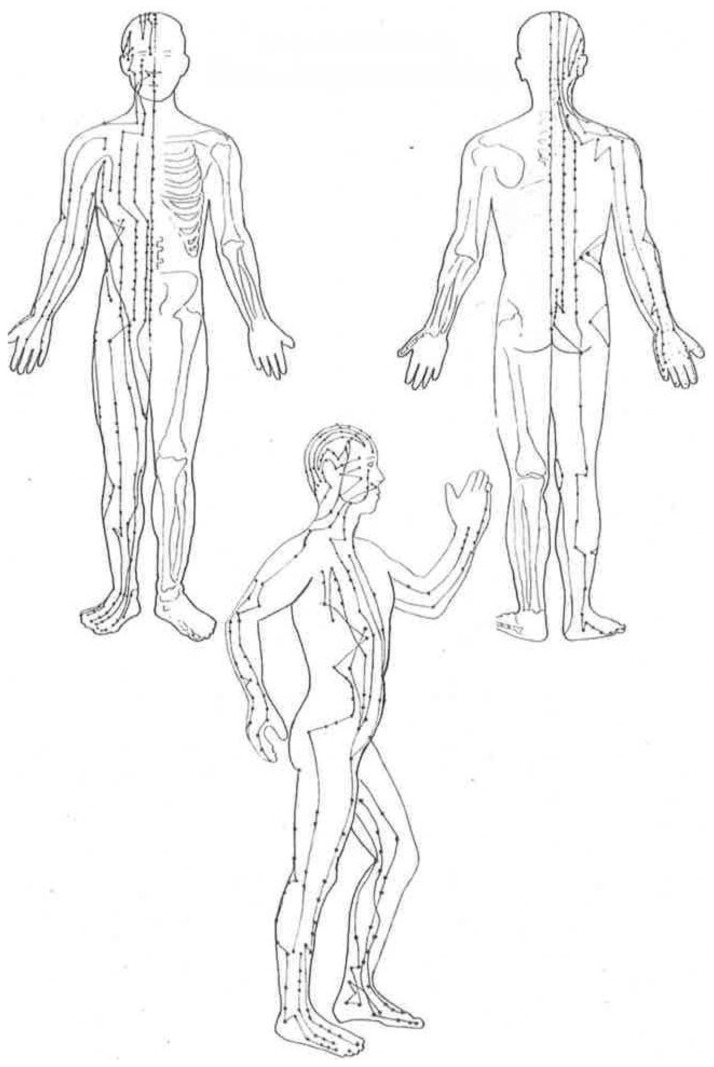
Meridians (lines); Acupuncutre points (dots) from the 12 major meridians. (Reproduced with permission from Picard, G. *Heal Yourself with Qigong*. Spiral Graphics: Canada; 2009; p. 47).

Qi circulation occurs through pathways of flow including 12 major meridians and 8 curious meridians [3]. Through modern science, schematics of the major meridians and the direction of flow paths have been generated by recording micro electrical differentials at points on the skin identified as acupuncture points [36] as illustrated in Figure 1. Stimulation of acupuncture points can occur through needling, electrolysis, laser, moxibustion, and acupressure [37]. Activation of acupuncture points has been shown to influence organ function and epigenetics (gene expression) [38]. While little is known of the mechanism of action of acupuncture point stimulation, effects in clinical applications are known to be as varied as management of asthma [39], kidney and liver function [40], pain [41], stress and anxiety [42], carpel tunnel syndrome [43], allergy [44], nausea [45], and weight loss [46]. Similar to acupuncture therapy, a proposed benefit of Qigong exercise is to maintain the patency of meridian paths through Qigong exercise and self-stimulation of acupuncture points. This self-stimulation occurs through processes such as internal muscle energy techniques that create mechanical forces generated through exercise movement and compression of soft tissue through fascial stretching. Whereas the goal of clinical acupuncture is to episodically correct and balance cyclic organ function and to promote the interdependence and healthful flow of energy (Qi), daily practice of Qigong exercise has the potential to create self-efficacy for continuous maintenance of homeostasis. 

In TCM, clinical treatment is approached with consideration that one has both a physical body and an energetic body or biofield that is both internal and external to the physical body. In addition to stimulation of acupuncture points within the physical body, adjustments to the energetic biofield can also influence ones health. While there has been little study of Qigong in terms of self-biofield adjustment and its effect on organ function, there is subjective evidence that external (applied by another) biofield massage such as Reiki or Therapeutic Touch can among other things, lessen fatigue, restore vitality, manage pain, and raise sense of well-being [47]. 

### 1.6. Physical: Western Medicine and Modern Exercise Science Theory

Functional, pain-free joint mobility requires skeletal stability, healthy articular cartilage, and appropriate extensibility of peri-articular dense connective tissues [48]. Articular cartilage is nourished by nutrients from the synovial fluid that are absorbed through the surface of the cartilage and nutrients that diffuse into the cartilage from the vascular subchondral bone [49,50,51]. Synovial fluid, which is both produced and reabsorbed by the synovial membrane that lines the joint capsule, provides a two-way transport of nutrients and clearance of wastes between cartilage and the blood stream. To effectively absorb nutrients and expel wastes, cartilage requires slow cyclical compression and decompression achieved by joint movement, muscle contraction, and intermittent weight bearing [52]. These conditions are hallmark features of Qigong movements. 

Basic human functional activities often require movement and coordination of multiple body parts and systems in multiple planes simultaneously. This is a common theme throughout Qigong and Tai Chi movements—the body moves as an integrated unit, through continuous, curved and spiral body movement patterns (multi-planar movements). Through these whole-body, continuous, circular movements, the fascial system of the body is engaged. According to the Fascia Research Congress, fascia is the soft tissue component of the connective tissue system that permeates the human body forming a whole-body continuous three-dimensional matrix of structural support [53]. It interpenetrates and surrounds all organs, muscles, bones and nerve fibers, creating a unique environment for body systems functioning. The scope of our definition and interest in fascia extends to all fibrous connective tissues, including aponeuroses, ligaments, tendons, retinacula, joint capsules, organ and vessel tunics, the epineurium, the meninges, the periostea, and all the endomysial and intermuscular fibers of the myofascia. It is widely accepted that the fascial system may additionally fulfill several other important functions in the body including (but not limited to) architectural/structural, neurological functions, biomechanical force transmission, morphogenesis, and cellular signal transmission [54]. The number of receptors in the fascia far outnumber those in the muscle and around the joint [55,56]. Within these mechanoreceptors, the majority of input comes from the interstitial receptors that are intimately connected to the autonomic nervous system. Stimulation of these intrafascial mechanoreceptors leads to an altered proprioceptive input to the central nervous system, which then results in a changed tonus regulation of motor units associated with this tissue. The result is relaxed, freer moving and more pliable tissue [57].

Physical activity improves cognitive function and brain plasticity [58]. Many activities of daily life involve the simultaneous performance of multiple tasks concurrently challenging motor and cognitive functions. In aging, the ability to perform multiple tasks common in daily living such as walking while engaged in a concurrent mental task (e.g., walking and talking) may become impaired [59]. The research of Eggenberger et al., provided insight into the beneficial effects of locomotor-cognitive dual task training for gait and posture performance and for processing speed and executive function [60]. Research suggests a causal relationship linking cognitively and physically demanding motor training, such as dance, to improvements in executive function [60]. Qigong practice integrates training in balance, flexibility, and neuromuscular coordination with a number of cognitive components including heightened body awareness, focused mental attention, imagery, multi-tasking, and goal-oriented training which together may result in benefits to gait health and postural control, beyond conventional uni-modal exercise [61]. Other research confirms a range of cognitive benefits gained from dedicated Qigong practice [62,63] 

The movements and practice of Qigong have characteristics that are beneficial throughout multiple systems of the body. Coordinated, spiral movements, often crossing midline are similar to complex movement patterns found in proprioceptive neuromuscular facilitation patterns used in neurological rehab. Neuroimmunolog*y* is the study of interactions among behavioral, neural, endocrine, and immunologic processes of adaptation [64]. Recent research has confirmed that Qigong practice has the benefits of mediating inflammatory response, raising immune support, and stimulating DNA repair [65]. Further discussion of neuro-integration and neurocognitive effects of MM can be found in the published work of Schmalzl et al. [66].

### 1.7. Purpose

The purpose of the current analysis is to explore mechanisms of action related to Qigong by unifying applied Eastern and Western exercise theory with the intention that it may serve as a model for future Qigong exercise analyses.

## 2. Methods

### 2.1. Design

The research is designed as an applied theoretical discussion of mechanisms of action for three selected Qigong exercises.

### 2.2. Research Team

Four experts in the field participated in this research. They include two physical therapists, a medical doctor with certification in Medical Acupuncture, and a Qigong Master of international reputation. All are certified Qigong instructors within the Wu Yi Jie He family system of Tai Chi and Qigong. All are experienced researchers in the field. Three have academic teaching affiliations. Together, they have over 56 years of cumulative Qigong practice. 

### 2.3. Exercise Selection Process

Criteria for selection of dynamic postures included: (a) application for three major body areas (upper body, trunk, and lower extremities); (b) application for common clinical problems (shoulder impairment, lower back and hip complaints, and balance training); (c) applications of neuro-motor integration; (d) a range of proposed mechanisms of therapeutic effect; and (e) referent for traditional postures that appear as derivative within multiple Qigong forms.

### 2.4. Data Extraction and Results

Three dynamic postures meeting selection criteria were identified from the modern 24-Movement Qigong Form [67] and are described and illustrated in the Results section of this discussion in preparation for applied theoretical analysis.

### 2.5. Data Analysis

Discrete analyses of mechanisms of action are based on consensus expert opinion of the researcher team. Sources include integration of available research, applied theory, and connoiseurial interpretation of Qigong as experienced by the researcher experts. By consensus thematic analysis, researchers identified major categories of action and subsections within those major categories. 

## 3. Results

**Figure 2:**
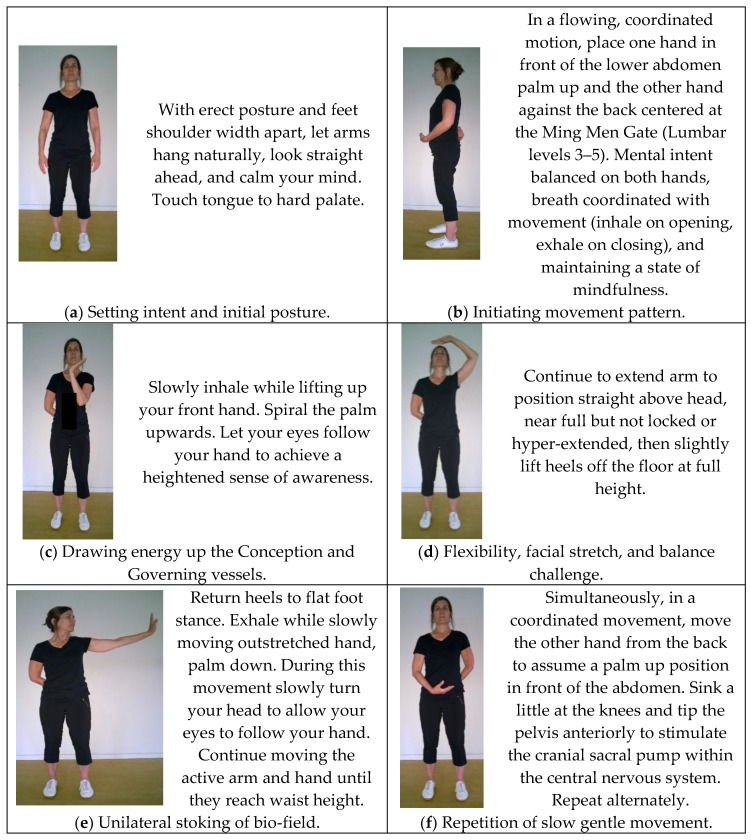
‘Plucking the Stars’. Intent and purpose of the exercise progression as well as performance instructions are provided within the illustrations.

**Figure 3:**
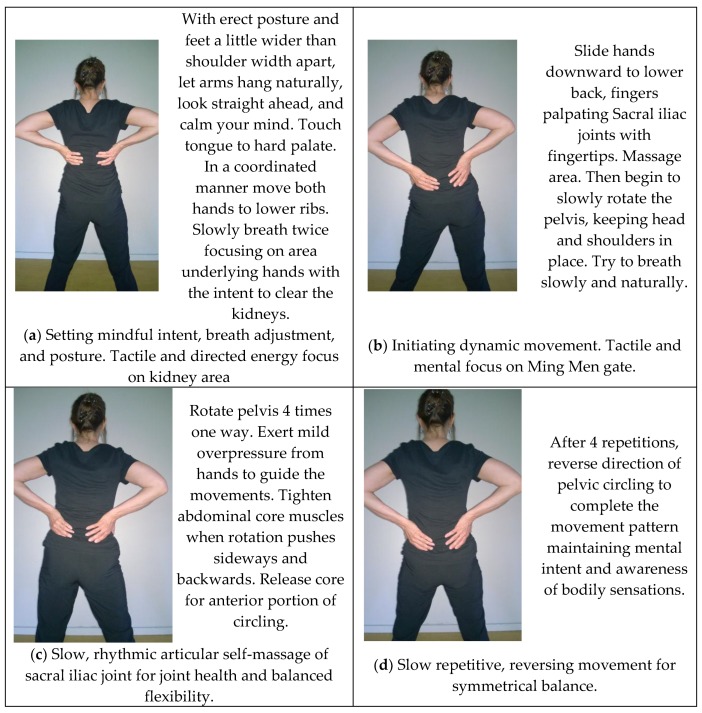
‘Lotus Leaves Rustle in the Wind’. Intent and purpose of the exercise progression as well as performance instructions are provided within the illustrations.

**Figure 4:**
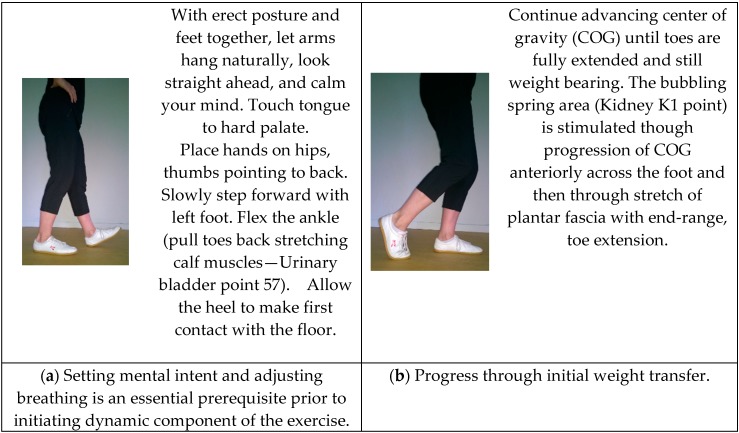

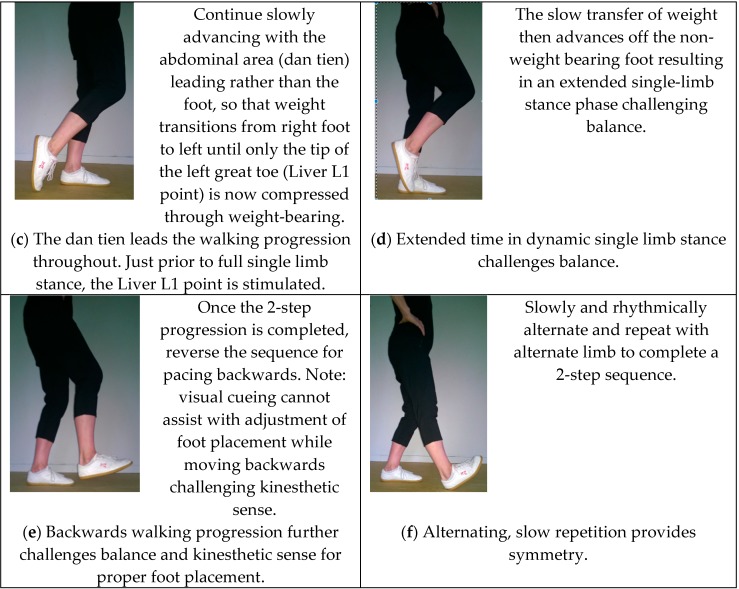
‘Pacing Forwards and Backwards’. Intent and purpose of the exercise progression as well as performance instructions are provided within the illustrations.

The three exercises selected for analyses were ‘Plucking the Stars’, ‘Lotus Leaves Rustle in the Wind’, and ‘Pacing Forwards and Backwards’ from the Wu Yi Jie He family system, 24-Posture Qigong form. This form, initially named *Twenty Exercises for Treating Diseases and Prolonging Life*, was codified in the 1950’s by Grand Master Wang Ziping, a doctor of TCM and esteemed martial arts master [68]. Partially based on martial arts, the form was designed as a complete, mind/body maintenance exercise system to prevent disease, build physique, delay senility, and prolong life and served as a precursor to modern physical therapy in China. The exercises embody the unity between respiration and mental regulation, partial and whole task, motion and stillness, movement and self-massage. Depictions of the original 20-movement form are available within a 1950’s text compiled by a panel Chinese Qigong masters. An English translation of that text was published in 1984 [68]. Figure 2, Figure 3 and Figure 4 illustrate key postures within dynamic movement progressions. A video demonstration is also available online as Supplemental data.

## 4. Analyses

The following discusses mechanisms of action related to meditative intent, energetic aspects, and physical aspects for each of the three selected exercises. Note that in the energetic body (biofield), all meridians are stimulated to one degree or another with any gross movement, however, depending on the specific exercise some meridians have more direct stimulation based on muscle contraction and fascial stretch generating mechanical forces acting on viscera and soft tissue including lymphatics. (See Table 1. for a summary of comparative aspects of three selected Qigong exercises.) 

**‘Plucking the Stars’**

### 4.1. Meditative Aspect

Preliminary to receiving instruction in motor performance for each of the selected exercises, practitioners are trained in essentials of meditation and meditative movement. Practioners are instructed to be aware of breath adjustment and both interoceptive and exteroceptive sensations throughout the exercises . In the latter half of the ‘Plucking the Stars’ movement, eyes follow the hand throughout the movement progression stroking the external bio-field. Practitioners are instructed to focus attention to facilitate an increased awareness of sensations at the fingertips, palm and the surface of the underarm during this part of the movement.

### 4.2. Energetic Aspect

Throughout each exercise, intent leads Qi (Yi Dao; Qi Dao. Mind leads energy flow). The reference source used in the linking of meridians to organs and organ function is a primary text on clinical acupuncture [37]. In ‘Plucking the Stars’, the upper arm and upper spine systems are most involved. The arms have 6 meridians, three Yin and three Yang. Yin Qi flows towards the fingers on the underside of the arm. Yang energy flows from the fingers towards the head on the outer side of the arm. Yin organs activated are the heart, pericardium and lungs. Yang organs activated are the small intestine, large intestine, and triple warmer (the energy center located superior and inferior to the diaphragm also known as the San Jiao). 

The following energetic analysis is based on application of energy theory. In the first part of the exercise as the active hand rises through midline, intent is simultaneously focused on both hands to stimulate micro-cosmic orbit or small heavenly circulation (xiao zhou tian) consisting of the Conception (du mai-yin highway) and Governing (chong mai-yang highway) vessels. The placement of the tip of the tongue on the hard palate connects the two vessels or pathways. The front hand slowly rises in midline in the path of the Conception vessel until the arm reaches full extension above the head. The upper bio-field (internal and external to the physical body) is combed at arm’s length throughout the return to resting position. Mental intent is maintained. This intent directs the path of the Qi flow. Through the lifting, spiraling, stretching, contracting and relaxing of soft tissue, any impedance to upper body energy flow is minimized. In returning the outstretched arm to the side, the bioelectric field is stroked with the hands and activated by mental intention assisted by eye focus. Simultaneously, the hand maintained at the back stimulates the Ming Men Gate of Vitality and the Governing vessel. The Governing vessel is one of the eight curious meridians and unites the Yang (Fu-hollow organs). Qi flows up the spine internally to the midline of the hard palate where it meets the Conception vessel. The Conception vessel is also categorized as a curious meridian and controls the Yin (Zang-solid organs) organs. It flows up the anterior midline to the center to the hard palate. Mediastinal structures and organs are stimulated by both movement and intention. The heart and its associated blood vessels are considered the root of life [69]. The alternating pattern facilitates energy balance symmetry. 

### 4.3. Physical Aspect

Analyzed from a biomechanical model, the movement through elongation or extension of the thoracic and cervical spines, with the arm progressively extending overhead, wrist extended. The thoracic and cervical spines are subject to losing mobility as we age, adapting more of a curved or rounded forward posture. This may also be exacerbated by various medical conditions (i.e., osteoporosis, spinal stenosis) and postures adapted during various activities (extended periods of sitting at a desk or driving). This lack of mobility may contribute to faulty or inefficient apical breathing patterns, loss of balance skill, and pain. This lack of mobility can also lead to dysfunction in the mechanoreceptors in your neck and back. Koskimies et al., (1997) reported that individuals with “tension neck”, had greater postural deviations induced by vibration of their neck than persons without a stiff neck. In other words, a tight neck might increase input from muscle proprioceptors, and dizziness due to too much proprioception [70]. Magnussen et al., (2006) similarly reported that when the neck is activated, cervical input is switched to become dominant over vestibular input [71]. One could hypothesize that in cervical vertigo could occur when neck input became dominant over vestibular, due to neck pain or stiffness. 

Another important feature of this complex posture is the extension of the arm over the head with the palm facing upwards, the wrist extended, and fingers pointing inward. Glenohumeral rhythm is maintained by keeping the hand close to midline and the shoulder joint in a loose-packed position minimizing the possibility of shoulder structure impingement as it rises. The act of assuming this position provides a gentle stretch of the nerves and surrounding fascia in the shoulder, arm, wrist, and hand, stimulating meridians of the arm and aiding in upper arm lymphatic drainage. This posture, along with extension of the spine, is beneficial for several reasons including breath adjustment and facilitation of CranioSacral pump. Training isolated to individual muscle groups may exercise that muscle, but can leave out integrated fascial tissues necessary to the body’s health in functional movement. Basic human functional activities often require complex, whole task movement with coordination of multiple body parts and systems in multiple planes [72]. This is a common theme throughout Qigong movements. The body is moving as an integrated unit, through multiple planes working every fabric of the body. Second, there are as many as 6 times the number of receptors in the fascia surrounding any given muscle than in the muscle itself [73]. This enables the fascia to provide more proprioceptive information to the brain and nervous system than is available from muscle.

**‘Lotus Leaves Rustle in the Wind’**

### 4.4. Meditative Aspect

In the first part of ‘Lotus Leaves Rustle in the Wind’, hand are placed over the kidney area as ribs expand with deep breathing there is an interoceptive awareness of the breath adjustment. In the second half of the exercise, hands are place on the Ming Men energy gate. Interoceptive and exterocepting (throught touch) awareness are focused on the mobility of the pelvic bones. 

### 4.5. Energetic Aspect

In ‘Lotus Leaves Rustle in the Wind’, intitially the hands are placed over the kidney area. Practitioners are instructed to imagine that each clearing breath energizes and clears the kidneys of any toxins. In the second part of the exercise. In the second part of the exercise, intention focused on opening of the Ming Men gate and visceral stimulation with intent to store (accumulate) renewable energy generated from the exercise into the lower Dan Tien vessel. Assuming a wider stance stabilizes the lower extremities and upper spine. Rotations occurring at the pelvis create a mechanical force on the Yin organs of the spleen, kidney and liver and the Yang organs of the urinary bladder, gall bladder and stomach. The spleen controls movement of the fluids in the stomach. Gall bladder is appended to the liver, and they mutually assist one another. The kidneys control water and filter toxins. The liver stores and transforms the blood and among its many functions has a role in regulation of blood pressure. The urinary bladder flushes fluids for elimination. The stomach assists in digestion. All Yin (Zang) and Yang (Fu) organs are replenished with Qi from the stomach. The rhythmic contraction and relaxation of trunk core, the gluteals, the muscles of the inner thigh, and internal pelvis stimulate several acupuncture points on the meridians flowing to and through the legs and hip areas.

### 4.6. Physical Aspect

This movement starts with the feet slightly wider apart than shoulder width, hands on the low back. Keeping the head and lower body relatively static, the pelvis is then rotated in a circular direction in one direction for 4-8 times, followed by the opposite direction with slight over pressure from the hands to assist in lateral shift. This rotation of the pelvis on the femur coupled with the contractions and relaxation of muscles of the trunk core and in and around the pelvis and thighs provide slow cyclical compression and decompression achieved by joint movement, muscle contraction, and intermittent weight bearing required to nourish cartilage in the joint. The slow, rhythmic rotation of the pelvis on the femur also helps in relaxation of the spinal and pelvic muscles [72]. 

**‘Pacing Forwards and Backwards’**

### 4.7. Meditative Aspect

In ‘Pacing Forwards and Backwards’, mental intent focuses on bodily sensations created by the slow, progressive transition of weight bearing from one foot to the other and the intention that the abdominal core (coinciding with the location of the lower Dan Tien) leads the slow, alternating, heel-to-toe, forwards and backwards walking progression. 

### 4.8. Energetic Aspect

In the mobility exercise: *Pacing Forwards and Backwards*, all lower leg meridians are activated. The weight-shift progresses laterally and from posterior to anterior and then reversing resulting in stimulation of Liver L1 point, Kidney K1 point and acupuncture points along the spleen meridian. Stabilizing of lateral-weight shift stimulates the gall bladder meridian and in particular: GB31 point located mid lateral thigh. Among other things, the slow reversing cycle of contracting and stretching the calf muscles acts on urinary bladder UB57 point. 

### 4.9. Physical Aspect

This movement starts with the feet together and the knees bent slightly. A majority of the movement is performed with both knees bent slightly elevating and lowering slightly in rhythm with weight shifting, producing an eccentric and concentric contractions through the quadriceps and gluteal muscles and CranioSacral pump. One of the legs is then advanced forward, contacting the ground with the heel first as weight is slowly transferred from one leg, to both, and then the alternate leg in a cyclic pattern. In this gait, walking is centered and controlled throughout, in contrast to a modern Western descriptions of gait as controlled falling. There is also a balance challenge resultant for the slow fluid pacing and increased proprioceptive and kinesthetic awareness associated with correct foot placement, while the body or center of mass (COM) remains unchanged. Next, the weight is slowly transferred forward and the person begins to shift weight onto the lead foot. This transition from double limb to single limb stance has been identified as the most hazardous phase for a slip during level walking [55]. As the weight is shifted over the lead foot, the rear foot slowly and with increased awareness goes onto the ball of the foot followed by extension of the great toe in a closed chain position. Slow, mindful practice of this movement allows the hallux to obtain the necessary extension (60°–65°) to transform the foot into a more rigid lever during gait, allowing the hallux and first metatarsal head to support normal weight-bearing loads facilitating flexor hallucis longus action for that muscle’s role in propulsion [74]. 

## 5. Discussion

The following discusses analyses by major category as well as challenges in application, suggestions for future research and limitations of the work.

For millennia, it was the convention to explain Qigong exercise in terms of TCM and energy theory. More recently modern research has explored the benefits of this ancient exercise within Western exercise physiology and integrative health care. A challenge of informing practice from a Western medicine perspective has been the lack of operational definition of Qigong exercise. The movement analysis conducted herein has identified several mechanisms of action suggesting that Qigong exercise is more than just slow, gentile, repetitive exercise. These mechanisms have been organized into three major categories: meditative, energetic, and physical aspects with subsections within each. Meditative aspects include relaxation response, interoception and exteroception. Energetic aspects include stimulation of meridians through acupressure, mental intent, and self-massage. Physical aspects include flexibility, strength, articular stimulation, neuro-integration, respiratory effect, fascial stretch, visceral massage, balance challenge CranioSacral pump, lymphatic and venous return and glandular stimulation, and physiologic response to relaxation. These major aspects and subcategories of action may serve to organize analysis of other dynamic Qigong exercises [75].

### 5.1. Meditative Aspects

As a mindfulness exercise, meditation is an essential element of Qigong. There is a strong body of evidence defining effects and mechanisms of action for meditation that generalizes to the meditative and mindful practices of Qigong. While related research is extensive and a full discussion of this topic is beyond the scope of this article, a brief discussion is warranted. Recent meta-analyses and individual studies demonstrated common brain effects for attention-based meditative practices and active-based meditations in the brain areas involved in reward processing and emotional control [76]. Hölzel et al., found that after 8-weeks of daily static meditative training, novice practitioners demonstrated brain plasticity. Whole brain analyses identified increases in the posterior cingulate cortex, the temporo-parietal junction, and the cerebellum in the MBSR group compared with the controls. The results suggest that participation in MBSR is associated with changes in gray matter concentration in brain regions involved in learning and memory processes, emotion regulation, self-referential processing, and perspective taking while deactivation was seen in the amygdala [77]. The amygdala, a part of the limbic system, is a neural structure associated with, among other things, emotions of fear, aggression and anxiety. This research is strong and implies the validity of ancient beliefs that meditative Qigong practice results in adjustments of the mind and more modern beliefs about how heightened awareness of sensations associated with Qigong practice can evolve into a deeper sense of self. All three exercises exhibit cardinal aspects of MM. Key for each of the three exercises discussed, practitioners are instructed to calm the mind, adjust breathing, and raise awareness of sensations (interoception and exteroception). 

### 5.2. Energetic Mechanisms

Unique to Qigong exercise among MM modalities is the intention to affect energy flow or clear Qi stagnation regionally and to mechanically stimulate acupuncture points. An example of this intentional aspect occurs in ‘Lotus Leaves Rustle in the Wind’, where hands are placed over the kidneys and there is instruction to practitioners to imagine that each breath clears the kidney to optimize organ function. 

### 5.3. Physical Mechanisms

Beyond articular stimulation, improved flexibility, and muscular coordination within the physical domain, the relaxation effect of slow deep breathing is evident in Qigong practice. In addition to this relaxation response and optimalization of respiratory function, the dynamic exercise within each of the three exercises discussed also includes facilitation of the CranioSacral Pump. The concept of CranioSacral Pump is the basis for Cranio/Sacral Therapy (CST). Cranio/Sacral Therapy is a gentle, hands-on method of evaluating and enhancing the functioning of a physiological body system called the craniosacral system—comprised of the membranes and cerebrospinal fluid that surround and protect the brain and spinal cord [78]. Self-regulation of the circulation of the cerebral spinal fluid is associated with the rhythmic and reciprocal changes in abdominal, thoracic and cerebral pressures and the lengthening and curving or shortening of the spine that occurs in normally with the ebb and flow of breathing. In Qigong exercise, this pumping action is accentuated through the classic sinking of center of mass occurring with softening of the knees with concomitant lengthening of spine by flattening spinal curve and anterior/posterior shifting of the pelvis that occurs with each exercise repetition. This pumping action helps the cerebral spinal fluid to circulate around the central nervous system (spinal cord and brain) promoting nutritional and tonifying filtering support for these neural structures.

### 5.4. An Example of Application Challenge

The third dynamic Qigong posture: ‘Pacing Forwards and Backwards’, is a dynamic balance training exercise. The evidence that dynamic Qigong and Tai Chi can improve balance and aid in fall prevention has been addressed in previous research. In the Central Sidney Tai Chi Trial (N = 702), relatively healthy older adults underwent a 16-wk program of community Tai Chi training [79]. This was a pragmatic study exerting little control over form choice and instructor competence. Results showed improvement in balance and incidence of falls among the experimental group without regard to style or form of Qigong exercise. Wayne et al., in a similar pragmatic RCT involving 60 healthy older adults, who receive 6 months of TC training, concluded that positive effects of short- and long-term TC were observed only under cognitively challenging duel task conditions and only for stride-time variability [80]. Similar to the Central Sidney study, other than frequency and duration of training, actual intervention protocols varied within participating community centers. Together, these two cardinal studies raise an interesting question: What element or elements do all of the Tai Chi styles and forms used in the studies have in common that can successfully result in improved balance and fall prevention? In related study (N = 25), Gou et al., found that Tai Chi Chih group performed better than the control group in balance, proprioception, and muscle strength of lower extremity [81]. In this study, researchers concluded that proprioception was considered the most important factor related to balance ability and accounted for explaining 44% of variance in medial-lateral sway direction, and 53% of variance in antero-posterior sway direction. These researchers concluded that proprioception may be an important factor affecting balance ability. It is likely that it is the gestalt of the complex system of Qigong exercise including heightened awareness, improved flexibility, proprioception, kinesthetic sense, and postural correction as well as whole task training such as slow pacing forwards and backwards that plays important roles in balance training and fall prevention. Hopefully, future research will aid in further understanding of these phenomena.

### 5.5. Future Research

The vast majority of research on therapeutic value of Qigong exercise in integrative health care addresses assessment of effect. It is only very recently that research agenda have begun to address mechanisms of action. Research on meditation is strong and infers benefit of MM where intent and learned relaxation combined with dynamic exercise are considered essential elements of the exercise phenomenon. Further research exploring similarities and differences between static and moving meditation may provide added insights for practice. 

Knowledge of physiologic and biomechanical actions occurring within Qigong practice is sufficiently documented to, at least, partially validate the proposition that slow, gentle, spiral movements result in mechanical stimulation of articular surfaces, soft tissues, and organs. While mapping of acupuncture points and meridians provide some evidence of the existence of an energy system within the body, and clinical research has also provided evidence of effect of acupuncture point stimulation, it is a jump to infer that Qigong exercise self-stimulates this energetic system. While the notion that muscle action and fascial stretch can result in electrical differential and chemical changes at acupuncture points similar to acupuncture therapy can be assessed, direct evidence of changes in Qi flow is not so easily measured. This may be due, in part, to limitations in instrumentation. Advances in micro-electric, infrared, magnetic, and vibrational bio measurement hold promise to expand our knowledge of mechanisms of action [82]. Until that time, theoretical knowledge of proposed mechanisms of action for specific Qigong exercises can offer some guidance for further research and future prescriptive integrative clinical practice. 

Another future research approach is to operationally define a set or individual Qigong exercise patterns and propose a theoretical model of mechanism of action on organs and structures. Predictions of how given Qigong exercises or forms might normalize organ function might then be used to assess validity of proposed theoretical models. Likewise, if it is purported that mental intent such as demonstrated in the second exercise: ‘Lotus Leaves Rustle in the Wind’, can improve kidney organ function, this could be assessed in individuals with abnormal function tests to determine if there is any normalization of kidney function with targeted Qigong practice.

In addition to quantitative theory validation, Dr. C. Kerr, a specialist in the field of brain and brain-body mechanisms underlying mindfulness and mind-body therapies including Tai Chi and Qigong, has suggested that understanding how one perceives Qigong exercise through ethnographic study may provide additional valuable information [83]. As sentient beings, we perceive and process our interaction with our world by complex means. A familiar example of response to our world is evident in the fight or flight reaction. We have a learned somatic stress response in anticipation of danger or stressful situations, such as the ringing of a telephone in the middle of the night. If an emergency is anticipated by the triggering of the ringing phone, then an instantaneous physiologic, somatic, emotional and cognitive stress reaction occurs. There may be many such stress triggers in a normal day of life, Activation of a learned stress-management strategy, one as instantaneous as the stress response, can assist to preserve health by countering the stress reaction. Using simple, rather than complex Qigong movement patterns to heighten sensing of breath, body and mind adjustment, such as slowing of heart rate, calming of the mind, release of endorphins is such a technique. This type of somatic-focused training can preface or be simultaneous with learning more complex pattern training and is integral to MM. Increased self-awareness may be important for more than stress management. For example, increased sensitivity to how we respond to activity can be used to guide energy conservation for those dealing with fatigue from cancer and other chronic condition. Research that provides insight into how novice vs. expert Qigong practitioners elicit and experience mindfulness can assist us to teach this skill more efficiently. Similarly, longitudinal research may provide information about learning progression. 

### 5.6. Limitations

An a priori limitation of this discussion is the limited availability of mechanistic research into how Qigong works. Therefore, much of the analysis is inferred application of theory rather than validated proof. As we learn more about mechanisms of action, this limitation will diminish. A potential limitation may be that a relatively small team of investigators conducted the dynamic posture analyses. However, the team does represent requisite expertise in both Eastern and Western medicine and therapeutic exercise, so any deficit in this area is considered minimal. There is also a potential bias in selection of the exercises chosen for analyses. Again, this bias is considered minimal as variations and derivations of these traditional Qigong exercises appear in numerous Qigong forms. 

## 6. Conclusions

Energetic, meditative, biomechanical, and physiologic analyses of three Qigong exercises demonstrate a theoretical model of how Qigong practice can selectively and holistically contribute to optimal function for health maintenance, healing and longevity. Meditative aspects include relaxation response, interoception and exteroception. Energetic aspects include stimulation of meridians through mental intent, acupressure and self-massage. Physical aspects include flexibility, strength, articular stimulation, neuro-integration, respiratory effect, fascial stretch, visceral massage, balance challenge CranioSacral pump, lymphatic and venous return and glandular stimulation, and physiologic response to relaxation. Knowledge of mechanisms of action for specific Qigong exercises can guide practice instruction, prescriptive practice addressing clinical health issues, future research agenda toward adoption of Qigong practice within integrative health care. The model of analysis demonstrated in this discussion is offered to assist in these endeavors.

## Figures and Tables

**Table 1:** Summary of comparative aspects of three Qigong exercises from the 24-Posture Qigong forms: ‘Plucking the Stars’, ‘Lotus Leaves Rustle in the Wind’, and ‘Pacing Forwards and Backwards’.

Concept	‘Plucking the Stars’	‘Lotus Leaves Rustle in the Wind’	‘Pacing Forwards and Backwards’
Meditative Aspect			
Relaxation response	Intent on exhale	Intent on exhale	Intent on exhale
Interoception and exteroception	Heightened awareness of internal and external sensations	Heightened awareness of internal and external sensations	Heightened awareness of internal and external sensations
Energetic Aspect Analysis			
Cultivating energy flow by stimulation of acupuncture points through muscle contracting, stretching and fascial compressing of soft tissue and organs. (In addition to general stimulation of all meridians)	Upper extremity and cervical and upper thoracic spine, internal organ meridians.	Internal visceral points: Urinary bladder UB57—mid calf, and additional points along the abdominal core, and gluteal areas.	Lower extremity including Urinary bladder UB57—mid calf when flexing the ankle; and additional points along in the lateral thigh and kidney and liver points within the foot
Mental Intent	Intent leads Qi: Eyes follow hand throughout the movement (Yi dao, Qi dao). through the Conception and Governing vessels and the biofield.	Clearing the kidneys and mobility of pelvic system. Opening of the Ming Men gate.	Intent to lead movement progression with the Dan Tien (located at center of body mass) rather than the foot or leg.
Self-massage	Biofield stroking	Hand placement over kidney area.	--
Physical Analysis			
Flexibility	Maintenance of gleno-humeral rhythm and shoulder range; Cervical rotation	Lower thoracic, costovertebral joints; sacroiliac joints.	Great toe extension; ankle; plantar fascia, heel cords.
Strength	Scapular stablizers; Shoulder movers.	Lateral costals; Spinal stablizers and core muscles.	Lower limb muscles involved in ankle righting, quadriceps, hip movers and stablizers, trunk core,
Articular stimulation	Shoulder capsule; cervical rotation.	Pelvic Sacroiliac joint; L3/4, L4/5, L5/S1; acetabulum rotates around the head of the femur	Foot joint mobility; gentle loading and unloading of all weight bearing joints.
Neuro-integration	Complex synergistic movement pattern.	Isolated body segment (pelvic rotation) movement pattern.	Complex, whole task synergistic movement pattern.
Cognitive effect	New learning; meditative mind/body practice; adjustment of the mind.	New learning; meditative mind/body practice; adjustment of the mind.	New learning; meditative mind/body practice; adjustment of the mind.
Respiratory effect	Improved PO_2_; decreased CO_2_.	Improved PO_2_; decreased CO_2_.	Improved PO_2_; decreased CO_2_.
Fascial stretch	Full body fascial stretch	Mild reciprocating stimulation of fascia supporting viscera.	Lower leg fascial stretch; plantar fascia stretch.
Visceral massage	Fascial stretch	Pelvic rotation	Pelvic rotation
Balance challenge	Rising on toes—foot/lower leg strength and anterior/posterior balance challenge; vestibular challenge in turning of the head; visual cueing.	Controlled anterior/posterior and lateral weight-shift.	Extended time in single limb stance; ankle righting challenge; foot placement challenge; foot as a mobile adaptor and stablizer.
CranioSacral pump	Rhythmic softening of the knees coordinated with pelvic tilting and breathing.	Rhythmic softening of the knees coordinated with pelvic tilting and breathing.	Rhythmic softening of the knees coordinated with pelvic tilting and breathing.
Lymphatic, venous return, glandular stimulation	Salivary gland stimulation; upper chest and axillary lymphatics; upper arm venous return.	Abdominal and Pelvic lymphatics	Abdominal core engagement for pelvic lymphatic return; calf muscle pump for lower leg venous return.
Physiologic response re: Relaxation effect (mind/body connection)	Vagal response; Changes in EEG; mental calm; sense of well-being.	Vagal response; Changes in EEG; mental calm; sense of well being.	Vagal response; Changes in EEG; mental calm; sense of well-being.

## Supplementary Materials

The following are available online at www.mdpi.com/2305-6320/4/4/69/s1, Video S1: Video of three Qigong exercises.

## References

[1] Lui T., Qiang X.M. (2010). Chinese Medical Qigon.

[2] Liang S.-Y., Wu W.C., Breiter-Wu D. (1997). Qigong Empowerment: A Guide to Medical, Taoist, Buddhist, and Wushu Energy Cultivation.

[3] Picard G. (2009). Heal Yourself with Qigong.

[4] Chodzko-Zajko W., Jahnke R. (2005). National Expert Meeting on Qigong and Tai Chi: Consensus Report.

[5] Yang G.-Y., Wang L.-Q., Ren J., Zhang Y., Li M.L., Zhu Y.T., Cheng Y.J., Li W.Y., Wayne P.M., Liu J.P. (2015). Evidence Base of Clinical Studies on Tai Chi: A Bibliometric Analysis. Scherer, R.W. Ed. PLoS ONE.

[6] Solloway M.R., Taylor S.L., Shekelle P.G., Miake-Lye I.M., Beroes J.M., Shanman R.M., Hemplel S. (2016). An evidence map of the effect of Tai Chi on health outcomes. Syst. Rev..

[7] Huang Z.G., Feng Y.H., Li Y.H., Lv C.S. (2017). Systematic review and meta-analysis: Tai Chi for preventing falls in older adults. BMJ Open.

[8] Kumar A., Delbaere K., Zijlstra G.A., Carpenter H., Iliffe S., Masud T., Skelton D., Morris R., Kendrick D. (2016). Exercise for reducing fear of falling in older people living in the community: Cochrane systematic review and meta-analysis. Age Ageing.

[9] Li F., Harmer P. (2015). Economic evaluation of a Tai JI Quan intervention to reduce falls in people with Parkinson disease, Oregon, 2008–20011. Pre. Chronic. Dis..

[10] Klein P.J., Schneider R., Rhoads C.J. (2016). Qigong in cancer care: A systematic review and construct analysis of Qigong therapy. Support. Care Cancer.

[11] Oh B., Butow P., Mullan B., Clarke S., Beale P., Pavlakis N., Kothe E., Lam L., Rosenthal D. (2010). Impact of medical Qigong on quality of life, fatigue, mood and inflammation in cancer patients: A randomized controlled trial. Ann. Oncol..

[12] Liu P., You J., Sun Y., He Y., Sit H., Jia L., Wong M., Xia Z., Zheng X., Wang Z. (2017). The efficacy of Guolin-Qigong on the mind-body health of Chinese women with breast cancer: A randomized controlled trial. Qual. Life Res..

[13] Irwin M.R., Olmstead R., Carrillo C., Sadeghi N., Nicassio P., Ganz P., Bower J.E. (2017). Tai Chi Chih Compared With Cognitive Behavioral Therapy for the Treatment of Insomnia in Survivors of Breast Cancer: A Randomized, Partially Blinded, Noninferiority Trial. J. Clin. Oncol..

[14] Zeng Y., Luo T., Xie H., Huang M., Cheng A.S. (2014). Health benefits of qigong or Tai Chi for cancer patients: A systematic review and meta-analyses. Complement. Ther. Med..

[15] Wang X.-Q., Pi Y.-L., Chen P.-J., Liu Y., Wang R., Li X., Chen B.-L., Zhu Y., Yang Y.-J., Niu Z.-N. (2016). Traditional Chinese Exercise for Cardiovascular Diseases: Systematic Review and Meta-Analysis of Randomized Controlled Trials. J. AM Heart Assoc..

[16] Xiong X., Wang P., Zhang Y. (2015). Qigong for hypertension: A systematic review Medicine (Baltimore). Medicine.

[17] Nery R.M., Zanini M., Ferrari J.N., Silva C.A., Farias L.F., Comel J.C., Belli K.C., da Silveira A.D., Santos A.C., Stein R. (2014). Tai Chi Chuan for Cardiac Rehabilitation in Patients with Coronary Arterial Disease. Arq. Bras. Cardiol..

[18] Gu Q., Wu S.J., Zheng Y., Zhang Y., Liu C., Hou J.C., Zhang K., Fang X.M. (2017). Tai Chi Exercise for Patients with Chronic Heart Failure: A Meta-analysis of Randomized Controlled Trials. Am. J. Phys. Med. Rehabil..

[19] Ngai S.P., Jones A.Y., Tam W.W. (2016). Tai Chi for chronic obstructive pulmonary disease. Cochrane Database Syst. Rev..

[20] Guo J.B., Chen B.L., Lu Y.M., Zhang W.Y., Zhu Z.J., Yang Y.J., Zhu Y. (2016). Tai Chi for improving cardiopulmonary function ad quality of life in patients with chronic obstructive pulmonary disease. Clin. Rehabil..

[21] Song R., Grabowska W., Park M., Osypiuk K., Vergara-Diaz G.P., Bonato P., Hausdorff J.M., Fox M., Sudarsky L.R., Macklin E. (2017). The impact of Tai Chi and Qigong mind-body exercises on motor and non-motor function and quality of life in Parkinson’s disease: A systematic review and meta-analysis. Parkinsonism Relat. Disord..

[22] Zhou J., Yin T., Gao Q., Yang X.C. (2015). A Meta-Analysis on the Efficacy of Tai Chi in Patients with Parkinson’s Disease between 2008 and 2014. Evid.-Based Complement. Altern. Med. eCAM.

[23] Xiang Y., Lu L., Chen W. (2017). Does Tai Chi relieve fatigue? A systematic review and meta-analysis of randomized controlled trials. PLoS ONE.

[24] Chan J.S.M., Ho R.T.H., Chung F.-F., Wang C.-W., Yao T.-W., Ng S.-N., Chan C.L.W. (2014). Qigong Exercise Alleviates Fatigue, Anxiety, and Depressive Symptoms, Improves Sleep Quality, and Shortens Sleep Latency in Persons with Chronic Fatigue Syndrome-Like Illness. Evid. Based Complement. Altern. Med..

[25] Wong A., Figueroa A., Sanchez-Gonzalez M.A., Son W.M., Chernykh O., Park S.Y. (2017). Effectiveness of Tai Chi on Cardiac Autonomic Function and Symptomatology in women with fibromyalgia: A randomized controlled trial. J. Aging Phys. Act..

[26] Lynch M., Sawynok J., Hiew C., Marcon D. (2012). A randomized controlled trial of qigong for fibromyalgia. Arthr. Res. Ther..

[27] Zou L., SasaKi J.E., Wang H., Xiao Z., Fang Q., Zhang M. (2017). A Systematic Review and Meta-Analysis Baduanjin Qigong for Health Benefit: Randomized Controlled Trials. Evid. Based Complement. Altern. Med..

[28] Huston P., McFarlane B. (2016). Health benefits of Tai Chi. What is the evidence?. Can. Fam. Phys..

[29] Larkey L., Jahnke R., Etnier J., Gonzalez J. (2009). Meditative movement as a category of exercise: Implications for research. J. Phys. Act. Health..

[30] Payne P., Crane-Godreau M.A. (2013). Meditative Movement for Depression and Anxiety. Front. Psychiatry.

[31] Benson H. (2009). The Relaxation Response.

[32] Pal G.K., Velkumary S. (2004). Effect of short-term practice of breathing exercises on autonomic functions in normal human volunteers. Indian J. Med. Res..

[33] Mourya M., Mahajan A.S., Singh N.P., Jain A.K. (2009). Effect of slow- and fast-breathing exercises on autonomic functions in patients with essential hypertension. J. Altern. Complement. Med..

[34] Sawynok J. (2016). Qigong, Parasympathetic Function and Fibromyagia. Fibrom Open Access..

[35] Tsakiris M., Tajadura-Jimenez A., Costantini M. (2011). Just a heartbeat away from one’s body: Interoceptive sensitivity predicts malleability of body-representations. Proc. R. Soc. B.

[36] Ahn A.C., Colbert A.P., Anderson B.J., Martinsen O.G., Hammerschiag R., Cina S., Wayne P.M., Lanagevin H.M. (2008). Electrical properties of acupuncture points and meridians: A systematic review. Bioelectromagnetics.

[37] Jayasuriya A. (2002). Clinical Acupuncture.

[38] Grolleau-Julius A., Ray D., Yung R.L. (2010). The Role of Epigenetics in Aging and Autoimmunity. Clin. Rev. Allergy Immunol..

[39] Lin H.C., Lin H.P., Yu H.H., Wang L.C., Lee J.H., Lin Y.T., Yang Y.H., Li P.Y., Sun W.Z., Chiang B.L. (2017). Tai-Chi-Chuan Exercise Improves Pulmonary Function and Decreases Exhaled Nitric Oxide Level in Both Asthmatic and Nonasthmatic Children and Improves Quality of Life in Children with Asthma. Evid. Based Complement. Altern. Med..

[40] Xia W.G., Zheng C.J., Zheng X., Wang J. (2017). Effects of “nourishing liver and kidney” acupuncture therapy on expression of brain derived neurotrophic factor and synaptophysin after cerebral ischemia reperfusion in rats. J. Huazhong Univ. Sci. Technol. Med..

[41] Yin C., Buchheit T.E., Park J.I. (2017). Acupuncture for chronic pain: An update and critical overview. Curr. Opin. Anaesthesiol..

[42] Schroeder S., Burnis J., Denton A., Krasnow A., Raghu T.S., Mathis K. (2017). Effectiveness of Acupuncture Therapy on Stress in a Large Urban College Population. Acupunct. Meridian Stud..

[43] Ural F.G., Öztürk G.T. (2017). The Acupuncture Effect on Median Nerve Morphology in Patients with Carpal Tunnel Syndrome: An Ultrasonographic Study. Evid. Based Complement. Altern. Med..

[44] McDonald J.L., Smith P.K., Smith C.A., Changli Xue C., Golianu B., Cripps A.W. (2016). Effect of acupuncture on house dust mite specific IgE, substance P, and symptoms in persistent allergic rhinitis. Ann. Allergy Asthma Immunol..

[45] Stoicea N., Gan T., Joseph N., Uribe A., Pandya J., Dalal R., Bergese S.D. (2015). Alternative Therapies for the Prevention of Postoperative Nausea and Vomiting. Front. Med..

[46] Zhang R.Q., Tan J., Li F.Y., Ma Y.H., Han L.X., Yang X.L. (2017). Acupuncture for the treatment of obesity in adults: A systematic review and meta-analysis. Postgrad. Med. J..

[47] Baldwin A.L., Trent N.L. (2017). An integrative review of scientific evidence for reconnective healing. J. Altern. Complement. Med..

[48] McKee P., Hannah S., Priganc V.W. (2012). Orthotic considerations for dense connective tissue and articular cartilage—The need for optimal movement and stress. J. Hand Ther..

[49] Mow V.C., Proctor C.S., Kelly M.A., Nordin M., Frankel V.H. (2001). Biomechanics of articular cartilage. Basic Biomechanics of the Musculoskeletal System.

[50] Cummings G.S., Tillman L.J., Currier D.P., Nelson R.M. (1992). Remodeling of dense connective tissue in normal adult tissues. Dynamics of Human Biologic Tissues.

[51] Salter R.B. (1994). The physiologic basis of continuous passive motion for articular cartilage healing and regeneration. Hand Clin..

[52] Salter R.B. (1993). Continuous Passive Motion (CPM): A Biological Concept for the Healing and Regeneration of Articular Cartilage, Ligaments, and Tendons: From Its Origination to Research to Clinical Applications.

[53] Findley T., Schleip R., Findley & R Schleip T.W. (2007). Introduction. Fascia Research: Basic Science and Implications for Conventional and Complementary Health Care.

[54] Schleip R., Findley T.W., Chaitow L., Huijing P.A. (2012). Fascia: The Tensional Network of the Human Body.

[55] Barker D., Barker D., McIntyre A. (2012). The morphology of muscle receptors. Handbook of Sensory Physiology, Vol III: Muscle Receptors.

[56] Schleip R. (2003). Fascial plasticity—A new neurobiological explanation. J. Bodyw. Mov. Ther..

[57] Ratey J.J., Loehr J.E. (2011). The positive impact of physical activity on cognition during adulthood: A review of underlying mechanisms, evidence and recommendations. Rev. Neurosci..

[58] Al-Yahya E., Dawes H., Smith L., Dennis A., Howells K., Cockburn J. (2011). Cognitive motor interference while walking: A systematic review and meta-analysis. Neurosci. Biobehav. Rev..

[59] Wollesen B., Voelcker-Rehage C. (2014). Training effects on motor-cognitive dual-task performance in older adults: A systematic review. EURAPA.

[60] Eggenberger P., Theill N., Holenstein S., Schumacher V., de Bruin E.D. (2015). Multicomponent physical exercise with simultaneous cognitive training to enhance dual-task walking of older adults: A secondary analysis of a 6-month randomized controlled trial with I-year follow-up. Clin. Interv. Aging.

[61] Wayne P.M., Manor B., Novak V., Costa M.D., Hausdorff J.M., Goldberger A.L., Ahn A.C., Yeh G.Y., Peng C.-K., Lough M. (2014). A systems biology approach to studying Tai Chi, physiological complexity and healthy aging: Design and rationale of a pragmatic randomized controlled trial. Contemp. Clin. Trials.

[62] Zheng G., Liu F., Li S., Huang M., Tao J., Chen L. (2015). Tai Chi and the protection of cognitive ability: A systematic review of prospective studies in healthy adults. J. Prev. Med..

[63] Tao J., Chen X., Egorova N., Liu J., Xue X., Wang Q., Zheng G., Li M., Hong W., Sun S. (2017). Tai Chi Chuan and Baduanjin practice modulates functional connectivity of the cognitive control network in older adults. Sci. Rep..

[64] Voss D.E., Ionta M.K., Myers J.W. (1985). Proprioceptive Neuromuscular Facilitation: Patterns and Techniques.

[65] Ho R.H., Chan M.P.H., Wang C.-W., Lau B.W.M., Therefore K.F., Yuen L.P., Sham J.S.T., Chan C.L.W. (2012). A randomized controlled trial of qigong exercise on fatigue symptoms, functioning, and telomerase activity in persons with chronic fatigue or Chronic Fatigue Syndrome. Ann. Behav. Med..

[66] Schmalzl L., Crane-Godreau M.A., Payne P. (2014). Movement-based embodied contemplative practices: Definitions and paradigms. Front. Hum. Neurosci..

[67] Wu H. A Life Dedicated to Marial Artsand Healin. Chi-Kung, Tai-Chi and Fan.

[68] People’s Medical Publishing House, Beijing, China (1987). The Chinese Way to a Long and Healthy Life.

[69] Cohen K.S. (1997). The Way of Qigong.

[70] Koskimies K., Sutinene P., Aaito H., Starck J., Toppila E., Kaksonen R., Ishizaki H., Alaranta H., Pyykkó I. (1997). Postural stability, neck proprioception and tension neck. Acta Otolarvngol. Suppl..

[71] Magnusson M., Anderson G., Gomez S., Johansson R., Mártensson A., Karlberg M., Fransson P.A. (2006). Cervical muscle afferents play a dominant role over vestibular afferents during bilateral vibration of neck muscles. J. Vestib. Res..

[72] Gracovetsky S. (1989). The Spinal Engine.

[73] Schleip R. (2003). Fascial plasticity—A new neurobiological explanation. J. Bodyw. Mov. Ther..

[74] Boissonnault W., Donatelli R. (1984). The Influence of Hallux Extension on the Foot during Ambulation. JOSPT.

[75] Voss D. (1985). Proprioceptive Neuromuscular Facilitation.

[76] Acevedo B.P., Pospos S., Lavretsky H. (2016). The Neural Mechanisms of Meditative Practices: Novel Approaches for Healthy Aging. Curr. Behav. Neurosci. Rep..

[77] Hölzel B.K., Carmody J., Vangel M., Congleton C., Yerramsetti S.M., Gard T., Lazar S.W. (2011). Mindfulness practice leads to increases in regional brain gray matter density. Psychiatry Res..

[78] Upledger J.E. (2008). CranioSacral Therapy: What It Is, How It Works.

[79] Voukelatos A., Cumming R.G., Lord S.R., Rissel C. (2007). A randomized, controlled trial of tai chi for the prevention of falls: The Central Sydney tai chi trial. J. Am. Geriatr. Soc..

[80] Wayne P.M., Hausdorff J.M., Lough M., Gow B.J., Lipsitz L., Novak V., Manor B. (2015). Tai Chi Training may Reduce Dual Task Gait Variability, a Potential Mediator of Fall Risk, in Healthy Older Adults: Cross-Sectional and Randomized Trial Studies. Front. Hum. Neurosci..

[81] Guo L.Y., Yang C.P., You Y.L., Chen S.K., Yang C.H., Hou Y.Y., Wu W.L. (2014). Underlying mechanisms of Tai-Chi-Chuan training for improving balance ability in the elders. Chin. J. Integr. Med..

[82] Wayne P.M. (2013). The Harvard Medical School Guide to Tai Chi.

[83] Kerr C. (2002). Translating “mind-in-body”: Two models of patient experience underlying a randomized controlled trial of qigong. Cult. Med. Psychiatry.

